# Relationships of Age at Menarche and Menopause, and Reproductive Year with Mortality from Cardiovascular Disease in Japanese Postmenopausal Women: The JACC Study

**DOI:** 10.2188/jea.16.177

**Published:** 2006-09-04

**Authors:** Renzhe Cui, Hiroyasu Iso, Hideaki Toyoshima, Chigusa Date, Akio Yamamoto, Shogo Kikuchi, Takaaki Kondo, Yoshiyuki Watanabe, Akio Koizumi, Yutaka Inaba, Akiko Tamakoshi, the JACC Study Group

**Affiliations:** 1 Department of Public Health Medicine, Graduate School of Comprehensive Human Science and Institute of Community Medicine, University of Tsukuba.; 2 Public Health, Department of Social and Environmental Medicine, Osaka University Graduate School of Medicine.; 3 Department of Public Health/Health Information Dynamics. Fields of Science, Program of Health and Community Medicine, Nagoya University Graduate School of Medicine.; 4 Department of Food Science and Nutrition, Faculty of Human Life and Environment, Nara Women’s University.; 5 Infectious Disease Surveillance Cancer, Infectious Disease Research Division, Hyogo Prefectural Institute of Public Health and Environmental Science.; 6 Department of Public Health, Aichi Medical University.; 7 Department of Epidemiology for Community Health and Medicine, Kyoto Prefectural University of Medicine Graduate School of Medical Sciences.; 8 Department of Health and Environmental Sciences, Graduate School of Medicine, Kyoto University.; 9 Department of Epidemiology and Environmental Health, Juntendo University School of Medicine.; 10 Department of Preventive Medicine/Biostatistics and Medical Decision Making, Field of Social Science, Program in Health and Community Medicine, Nagoya University Graduate School of Medicine.

**Keywords:** Coronary Disease, Cerebrovascular Disorders, Menopause, Menarche, Follow-Up Studies

## Abstract

**BACKGROUND:**

Early menopause is associated with increased risk of coronary heart disease in Caucasian women. However, this association has not been examined in Asian women.

**METHODS:**

We conducted a 10-year cohort study of 37,965 Japanese post-menopausal women aged 40-79 years in the Japan Collaborative Cohort (JACC) Study. Causes of death were determined based on the International Classification of Disease.

**RESULTS:**

There were 487 mortality of stroke and 178 mortality of coronary heart disease. Late menarche or early menopause, or shorter duration of reproductive period was not associated with risk of mortality from coronary heart disease. However, compared with women with age at menarche ≤13 years, those with age at menarche ≥17 years tended to have increased risk of mortality from stroke: the multivariable hazard ratio was 1.32 (95% confidence interval [CI]: 0.93-1.87, p = 0.10). Compared with women with age at menopause of ≥49 years, those with age at menopause of <49 years tended to have increased risk of coronary heart disease among women aged 40-64 years; the multivariable hazard ratio was 1.85 (95% CI: 0.92-3.73, p = 0.08).

**CONCLUSIONS:**

The possible association between early menopause and coronary heart disease among middle-aged women was consistent with the result of observational studies for Caucasian women, and can be explained by a protective effect of endogenous estrogen on the development of atherosclerosis.

Several prospective studies have indicated that women who experience natural menopause at an early age have a higher risk of coronary heart disease;^1^^-^^4^ however, the relation between exogenous estrogen use for post-menopausal women and risk of coronary heart disease are controversial. Exogenous estrogen use for post-menopausal women has been associated with reduced risk of coronary heart disease.^5^^-^^7^ On the other hand, clinical trials demonstrated no benefit of hormone replacement therapy on risk of coronary heart disease.^8^^,^^9^ A recent prospective study has indicated that age at natural menopause was unrelated to stroke mortality.^10^ These findings are based on studies in Caucasian women, and no study has examined the potential effect of menstrual variables on the risk of coronary heart disease among Asian women.

Previous prospective studies indicated that serum total cholesterol levels were higher in post-menopausal women and in women on hormone replacement therapy than in premenopausal women.^11^^,^^12^ Endothelial dysfunction is pronounced after menopause possibly due to the reduction of endogenous estrogen.^13^ Using urinary cGMP excretion, a second messenger of nitric oxide, to estimate endothelial function, we have reported that nitric oxide bioactivity declined with higher serum total cholesterol levels in a general population; this relationship was more evident among post-menopausal women.^14^ Our *a priori* hypothesis was that early menopause and shorter duration of reproductive year are associated with an increased risk of mortality from coronary heart disease, and that these associations are more pronounced in younger age groups at baseline.

A large prospective cohort study with 10 years of follow-up was used to examine the relationship between a broad range of age at menarche, age at menopause, and duration of reproductive period with mortality from stroke, coronary heart disease, and total cardiovascular disease among Japanese post-menopausal women.

## METHODS

The Japan Collaborative Cohort Study for Evaluation of Cancer Risk Sponsored by Monbusho (JACC Study) began in 1988-1990, when 110,792 individuals (46,465 men and 64,327 women) aged 40-79 years living in 45 communities across Japan participated in municipal health screening examinations and completed self-administered questionnaires about their lifestyles and medical histories, and women were also asked for age at menarche, age at menopause, and type of menopause.^15^ Late menarche was defined as the age at menarche ≥17 years and early menopause was defined as the age at menopause ≤44 years. Duration of reproductive year was defined as the number of years between age at menarche and menopause. Informed consent was obtained from these individuals when they completed the questionnaire. Follow-up surveys were conducted annually to verify the vital status of the participants. We excluded 23,785 premenopausal women, and 2,577 women who had a history of stroke, coronary heart disease or cancer at baseline. Therefore, 37,965 women were enrolled in the present study.

### Baseline Surveillance of Mortality from Cardiovascular Disease

Follow-up surveys were conducted annually to determine the vital status of the participants, and the investigators conducted systematic review of death certificates, all of which were forwarded to the public health center in the area of residency. Mortality data were sent centrally to the Ministry of Health and Welfare, and the underlying cause of deaths was coded according to the International Classification of Diseases (ICD), 9th Revision from 1988 through 1994, and 10th Revision from 1995 through 1999 for the National Vital Statistics. Registration of death is required by the Family Registration Law in Japan and is believe to be followed across Japan. Therefore, all deaths that occurred in the cohort were ascertained by death certificates from public health centers, except for subjects who died after they had moved from their original community, in which case the subject was treated as a censored case. The follow-up was conducted until the end of 1999, and the average follow-up period of time for the participants was 10.0 years. The Ethics Committee of the University of Tsukuba approved the present study in advance.

Cause-specific mortality of cardiovascular disease was determined based on the ICD-9th revision and ICD-10th revision as follows: total cardiovascular disease (ICD-9th revision, codes 390 to 459, ICD-10th revision, codes I01 to I99), coronary heart disease (410 to 414, I20 to I25), total stroke (430 to 438, I60 to I69), and stroke subtypes such as subarachnoid hemorrhage (430 and I60), intraparenchymal hemorrhage (431 and I61), and ischemic stroke (433 and I63).

### Statistical Analyses

Statistical analyses were based on mortality rates of stroke, coronary heart disease and total cardiovascular disease during the follow-up from 1988-90 through 1999. For each participant, follow-up was calculated from the date of filling out the baseline questionnaire through time of death, moving out of the community, or the end of 1999, whichever was first. The hazard ratio of mortality from cardiovascular disease was defined as the death rate among participants in categories of age at menarche (≤13, 14, 15, 16, and ≥17 years), age at menopause (≤44, 45-46, 47-48, 49-50, and ≥51 years), and duration of reproductive year (≤27, 28-30, 31-33, 34-36, and ≥37 years). We used categories of age at menarche ≤13 years, age at menopause ≥51 years, and duration of reproductive period ≥37 years as the reference. The category of early age at menarche and menopause or shorter duration of reproductive period was defined as the approximately lowest deciles.

Age-adjusted means and proportions of selected cardiovascular risk factors and psychological factors were presented among the categories of age at menarche, age at menopause, and duration of reproductive period, using analysis of covariance or chi-square tests. Testing for a linear trend across the age at menarche, age at menopause, and duration of reproductive period categories was conducted by linear regression or logistic regression model, using a median variable of age at menarche, age at menopause, and duration of reproductive period in each category. The age- and multivariable-adjusted hazard ratios and the 95% confidence intervals (CIs) were calculated after adjustment for age and potential confounding factors by using the Cox proportional hazards model. The confounding variables included smoking status (never, ex-, current 1-19, and ≥20 cigarettes/day), alcohol intake categories (never, ex-, current ethanol 1-22, 23-45, 46-68, and ≥69 g/day), marital status (married, widowed, divorced, single), type of menopause (natural, surgical, or unknown), education (primary school, junior high school, high school, college or more), histories of hypertension (no, yes) and diabetes (no, yes). The analysis was repeated stratified by baseline age-subgroup (age at 40 to 64 years and age at 65 to 79 years).

## RESULTS

During the 10-year follow-up of 37,965 post-menopausal women aged 40-79 years, 1,010 women died of total cardiovascular disease. These deaths included 487 from stroke including 111 subarachnoid hemorrhages, 99 intraparenchymal hemorrhages, 167 ischemic stroke, and 178 from coronary heart disease.

Table 1 shows mean age and age-adjusted mean values and prevalence of selected cardiovascular risk factors by five categories of age at menarche and menopause, and duration of reproductive period. Women with age at menarche ≥17 years were older, smoked more, were less hypertensive and diabetic, had lower mean body mass index and lower education level compared with those with lower age at menarche categories. Women with age at menopause ≥51 years were older and more hypertensive, had a higher level of education, and were more likely to be married, but smoked less compared with those in lower age at menopause categories. Women with a longer duration of reproductive period were older and more hypertensive, had higher mean body mass index, and were higher education level, and were more likely to be married, but smoked less.

**Table 1.** Age-adjusted characteristics of 37,965 women aged 40-79 years

	Age at menarche (year)					

	≤13	14	15	16	≥17	P for trend
No. at risk	5,595	7,336	8,578	7,387	9,069	
Age (average, year)	59.1	60	61.1	62.8	63	<0.001
Body mass index (average, kg/m^2^)	23.3	23	22.9	23	22.8	0.05
Smoker (%)	4.7	4.1	4.4	5.1	5.5	<0.001
History of hypertension (%)	29.7	27.7	28	27.7	25.9	<0.001
History of diabetes (%)	5.9	5.4	4.3	4.3	4	<0.001
Ethanol intake (average, g/day)	10	9.3	10	10	10.1	0.59
College or higher education (%)	11	9.6	8.8	8.5	7.8	<0.001
Married (%)	77.8	79.6	79.6	81.1	80.8	<0.001

	Age at menarche (year)					

	≤44	45-46	47-48	49-50	≥51	
	
No. at risk	5,084	3,975	6,274	10,209	12,423	
Age (average, year)	59.7	61.7	61.4	61.9	61.7	<0.001
Body mass index (average, kg/m^2^)	22.9	22.7	22.9	22.9	23.1	0.06
Smoker (%)	6.7	5.1	5	4.4	4.1	<0.001
History of hypertension (%)	27.2	24.6	26.9	27.8	29	<0.001
History of diabetes (%)	5.6	4.5	4.4	4.4	4.8	0.63
Ethanol intake (average, g/day)	12	9.9	9.2	9.4	9.7	0.001
College or higher education (%)	8.4	8.1	7.9	8.8	10.2	<0.001
Married (%)	76.8	78.6	79.3	80.4	81.5	<0.001

	Duration of reproductive period (year)					

	≤27	28-30	31-33	34-36	≥37	
	
No. at risk	4,204	4,387	8,298	11,731	9,345	
Age (average, year)	60.6	61.5	61.7	61.3	61.6	<0.001
Body mass index (average, kg/m^2^)	22.9	22.7	22.9	22.9	23.3	0.03
Smoker (%)	6.7	5.8	4.9	4.4	3.8	<0.001
History of hypertension (%)	26.4	25.2	27	27.8	29.8	<0.001
History of diabetes (%)	5.5	4.2	4.2	4.6	5.3	0.17
Ethanol intake (average, g/day)	12.2	10	9.7	9.2	9.8	0.002
College or higher education (%)	7.9	8.4	7.5	9.1	10.8	<0.001
Married (%)	78	77.9	78.8	81	81.1	<0.001

The study began in 1988 to 1990 at baseline, followed until the end of 1999.

Table 2 shows age- and multivariable-adjusted hazard ratios of mortality from stroke, coronary heart disease, and total cardiovascular disease according to age at menarche and menopause, and duration of menstruation. Women with age at menarche ≥17 years had tented to increase risk of mortality from stroke; the respective multivariable hazard ratio was 1.32 (95% CI: 0.93-1.87, p = 0.10). The risk of mortality from coronary heart disease and total cardiovascular disease was not significantly increased among women with later ages at menarche. Also, women with later ages at menarche were not at an increased risk of mortality from intraparenchymal hemorrhages, subarachnoid hemorrhages, or ischemic stroke (not shown in the table).

**Table 2.** Hazard ratios (HRs) and 95% confidence intervals (CIs) of mortality from cardiovascular disease according to age at menarche, menopause, and duration of reproductive period.

	Age at menarche (year)				

	≤13	14	15	16	≥17
Person-years	55,608	73,075	85,672	73,992	90,747

	Total stroke deaths				
No.	42	83	90	127	145
Age-adjusted HR (95% CI)	1.00	1.28 (0.89-1.86)	1.04 (0.72-1.50)	1.46 (1.03-2.07)	1.36 (0.96-1.92)
Multivariable HR (95% CI)	1.00	1.29 (0.89-1.88)	1.03 (0.71-1.49)	1.42 (1.00-2.02)	1.32 (0.93-1.87)

	Coronary heart disease deaths				
No.	18	21	45	36	58
Age-adjusted HR (95% CI)	1.00	0.74 (0.39-1.38)	1.15 (0.67-1.99)	0.91 (0.51-1.60)	1.19 (0.70-2.03)
Multivariable HR (95% CI)	1.00	0.77 (0.41-1.45)	1.22 (0.70-2.11)	0.98 (0.55-1.73)	1.28 (0.75-2.20)

	Total cardiovascular disease deaths				
No.	96	165	212	230	307
Age-adjusted	1.00	1.11 (0.86-1.42)	1.05 (0.82-1.34)	1.13 (0.89-1.43)	1.23 (0.97-1.54)
Multivariable HR (95%CI)	1.00	1.13 (0.88-1.45)	1.06 (0.83-1.35)	1.13 (0.89-1.44)	1.22 (0.96-1.53)

	Total stroke deaths				

	≤44	45-46	47-48	49-50	≥51
	
Person-years	50,463	39,803	62,599	102,134	124,094

	Total stroke deaths				
No.	67	54	104	129	133
Age-adjusted HR (95% CI)	1.19 (0.89-1.60)	1.07 (0.78-1.47)	1.40 (1.08-1.81)	1.03 (0.81-1.32)	1.00
Multivariable HR (95% CI)	1.21 (0.89-1.64)	1.08 (0.78-1.49)	1.38 (1.07-1.79)	1.01 (0.80-1.29)	1.00

	Coronary heart disease deaths				
No.	23	16	33	40	66
Age-adjusted HR (95% CI)	0.80 (0.50-1.30)	0.62 (0.36-1.08)	0.87 (0.57-1.33)	0.63 (0.42-0.93)	1.00
Multivariable HR (95% CI)	0.78 (0.47-1.29)	0.63 (0.36-1.09)	0.87 (0.57-1.33)	0.62 (0.42-0.92)	1.00

	Total cardiovascular disease deaths				
No.	139	111	190	265	305
Age-adjusted HR (95% CI)	1.07 (0.88-1.32)	0.96 (0.77-1.19)	1.12 (0.93-1.34)	0.92 (0.78-1.09)	1.00
Multivariable HR (95% CI)	1.08 (0.88-1.34)	0.97 (0.78-1.21)	1.10 (0.92-1.32)	0.91 (0.77-1.07)	1.00

	Duration of reproductive period (year)				

	≤27	28-30	31-33	34-36	≥37
	
Person-years	41,725	43,941	83,127	117,275	93,026

	Total stroke deaths				
No.	55	68	143	120	101
Age-adjusted HR (95% CI)	1.08 (0.77-1.50)	1.19 (0.87-1.62)	1.36 (1.06-1.76)	0.88 (0.68-1.18)	1.00
Multivariable HR (95% CI)	1.07 (0.77-1.51)	1.18 (0.86-1.61)	1.33 (1.03-1.72)	0.86 (0.66-1.13)	1.00

	Coronary heart disease deaths				
No.	20	23	39	50	46
Age-adjusted HR (95% CI)	0.83 (0.49-1.41)	0.85 (0.52-1.41)	0.79 (0.51-1.21)	0.79 (0.53-1.18)	1.00
Multivariable HR (95% CI)	0.84 (0.49-1.45)	0.86 (0.52-1.42)	0.81 (0.53-1.24)	0.80 (0.54-1.20)	1.00

	Total cardiovascular disease deaths				
No.	119	132	265	275	219
Age-adjusted HR (95% CI)	1.06 (0.85-1.33)	1.05 (0.85-1.31)	1.16 (0.97-1.38)	0.93 (0.78-1.11)	1.00
Multivariable HR (95% CI)	1.07 (0.85-1.35)	1.05 (0.85-1.31)	1.15 (0.96-1.38)	0.93 (0.78-1.11)	1.00

Multivariable adjustment: age, body mass index (kg/m^2^), histories of hypertension and diabetes, current smoking, ethanol intake, marital status, college or higher school, and type of menopause.

Categories of age at menarche ≤13 years, age at menopause ≥51 years, and duration of reproductive period ≥37 years as the reference groups.

The study began in 1988-1990 at baseline, followed until the end of 1999.

Early menopause was not associated with the higher risk of mortality from stroke, coronary heart disease, or total cardiovascular disease. No significant association was observed between the duration of menstruation and mortality from stroke, coronary heart disease, and total cardiovascular disease. The proportion of menopause by surgery was 8.5% (3,242/37,965) among total subjects. When we excluded them from the analyses, the results did not change materially (not shown in the table).

The associations between age at menopause and coronary heart disease were further examined when stratified by age at baseline survey (Table 3). We found no excess risk of mortality associated with early menopause in either subgroup of ages 40-64 and 65-79 years. However, compared to women with age at menopause ≥49 years, those with age at menopause <49 years tended to have increased risk of mortality from coronary heart disease in the age subgroup of 40-64 years, but not in the older ages: the multivariable hazard ratio was 1.85 (95% CI: 0.92-3.73, p = 0.08) and 0.84 (95% CI: 0.60-1.19, p = 0.32), respectively.

**Table 3.** Multivariable hazard ratios (HRs) and 95% confidence intervals of mortality from coronary heart disease according to age at menopause stratified by age at the baseline survey.

	Age at menopause (year)				

	≤44	45-46	47-48	49-50	≥51
	Age at baseline survey = 40-64 years				
No	6	4	8	6	11
Multivariable HR	1.82 (0.61-5.41)	1.49 (0.47-4.75)	1.61 (0.64-4.07)	0.72 (0.27-1.97)	1.00

	Age at baseline survey = 65-79 years				
No	17	12	25	34	55
Multivariable HR	0.65 (0.37-1.15)	0.51 (0.27-0.96)	0.74 (0.46-1.19)	0.59 (0.39-0.91)	1.00

Multivariable adjustment: age, body mass index (kg/m^2^), history of hypertension and diabetes, current smoking, ethanol intake, marital status, college or higher school, and type of menopause.

The study began in 1988-1990 at baseline, followed until the end of 1999.

## DISCUSSION

In this large prospective study of Japanese menopausal women, late age at menarche tended to be associated with increased risk of mortality from stroke among total subjects aged 40 to 79 years, and early menopause tended to be associated with increased risk of mortality from coronary heart disease among younger ages of 40 to 64 years.

Mechanisms for the possible association between late menarche and stroke are not clear at present. Women with late age at menarche were older, smoked more, and were less educated in the present study. These characteristics have been associated with risk of stroke among Japanese.^16^^-^^20^ One-year case-fatality of ischemic stroke was 2-fold higher for Finnish women with lower income and 3-fold higher in those with lower education than those with higher socioeconomic status.^21^ A follow-up study showed that women with late age at menarche were likely to have lower body mass index and to be less obese among women aged 45 to 52 years.^22^ Further, our recent prospective study indicated that women with body mass index <18.5 kg/m^2^ had 2-fold higher risk of mortality from total stroke.^23^ Other potential confounding socioeconomic conditions such as income levels, which were not examined in the present study, may explain the association. Alternatively, the association could be due to chance.

Previous case-control studies reported that age at menarche (<13 vs. ≥13 years, or <15 vs. ≥15 years old) was associated with the 2-to 3-fold higher prevalence of subarachnoid hemorrhage,^24^^,^^25^ and the 5-fold higher prevalence of intraparenchymal hemorrhage.^25^ However, the present study showed that earlier age at menarche was not significantly associated with the risk of mortality form subarachnoid hemorrhage or intraparenchymal hemorrhage.

Early menopause tended to increase the risk of mortality from coronary heart disease among post-menopausal women aged 40 to 64 years, but not among those aged 65 to 79 years in the present study. This result is consistent with the finding that early menopause was associated with higher risk of mortality from coronary heart disease among American and European women.^1^^,^^2^^,^^26^ In studies of Norwegian and Dutch women, this association was more evident in women of younger ages than those of older ages.^1^^,^^26^ One of the reasons for the larger impact of early menopause at younger ages may be a longer duration of elevated serum total or LDL-cholesterol levels along with endogenous estrogen depletion.^27^ Depletion of estrogen itself may have an adverse effect of the development of atherosclerosis due to endothelial dysfunction^13^^,^^28^ and increased platelet aggregability.^29^

Observational studies have reported that the use of hormone replacement therapy was associated with reduced risk of coronary heart disease.^5^^-^^7^ Clinical trials, however, demonstrated no benefit of hormone replacement therapy on the risk of coronary heart disease.^8^^,^^9^ However, a potential benefit of estrogen therapy was found for young postmenopausal women aged 50-59 years.^9^

In the Nurses Health Study, early menopause was associated with a higher risk of coronary heart disease among current smokers, but not among never-smokers.4 This interaction was not found in the present study, where a very low prevalence (5%) of current smoking in our cohort made it difficult to evaluate the interaction reliably.

The strengths of the present study include its prospective design and large sample size. The limitations are that first we used the self-report of age at menarche and menopause. We did not test the reliability of these variables. However, previous studies using two-year repeated questionnaires showed that 81 to 88% of post-menopausal women reported concordant responses for age at menarche^30^ and for age at menopause.^30^^,^^31^ Second, we excluded 23,785 premenopausal women at baseline: 94.3% of these women were aged 40-64 years. Thus, for the younger age group, women with an early menopause would be over represented in the data set analyzed. This, however, is unlikely to introduce a serious bias in the evaluation of association between age at menopause and cardiovascular disease because the actual distribution of exposure variables may be irrelevant for the evaluation.

Mechanisms for the association between late menarche and mortality from stroke are uncertain, and could be due to chance. The possible association between early menopause and coronary heart disease among young women in the present study was consistent with the results in Caucasian women, which can be explained by a protective effect of endogenous estrogen on the development of atherosclerosis.
